# Electrical Properties of Low-Temperature Processed Sn-Doped In_2_O_3_ Thin Films: The Role of Microstructure and Oxygen Content and the Potential of Defect Modulation Doping

**DOI:** 10.3390/ma12142232

**Published:** 2019-07-11

**Authors:** Getnet Kacha Deyu, Jonas Hunka, Hervé Roussel, Joachim Brötz, Daniel Bellet, Andreas Klein

**Affiliations:** 1 Electronic Structure of Materials, Department of Materials and Earth Sciences, Technische Universität Darmstadt, Otto-Berndt-Straße 3, 64287 Darmstadt, Germany; 2 Univ. Grenoble Alpes, CNRS, Grenoble INP, LMGP, 38 000 Grenoble, France; 3 Structural Research, Department of Materials and Earth Sciences, Technische Universität Darmstadt, Otto-Berndt-Straße 3, 64287 Darmstadt, Germany

**Keywords:** ITO, electrical properties, doping limits, modulation doping, thickness dependence, low-temperature-deposition

## Abstract

Low-temperature-processed ITO thin films offer the potential of overcoming the doping limit by suppressing the equilibrium of compensating oxygen interstitial defects. To elucidate this potential, electrical properties of Sn-doped In${}_{2}$O${}_{3}$ (ITO) thin films are studied in dependence on film thickness. In-operando conductivity and Hall effect measurements during annealing of room-temperature-deposited films, together with different film thickness in different environments, allow to discriminate between the effects of crystallization, grain growth, donor activation and oxygen diffusion on carrier concentrations and mobilities. At $200{\;}^{\circ }C$, a control of carrier concentration by oxygen incorporation or extraction is only dominant for very thin films. The electrical properties of thicker films deposited at room temperature are mostly affected by the grain size. The remaining diffusivity of compensating oxygen defects at $200{\;}^{\circ }C$ is sufficient to screen the high Fermi level induced by deposition of Al${}_{2}$O${}_{3}$ using atomic layer deposition (ALD), which disables the use of defect modulation doping at this temperature. The results indicate that achieving higher carrier concentrations in ITO thin films requires a control of the oxygen pressure during deposition in combination with seed layers to enhance crystallinity or the use of near room temperature ALD.

## 1. Introduction

Transparent conductive oxides (TCOs) are key materials for electrodes in display and solar cell technologies [1,2,3,4,5,6]. The most prominent TCO materials are Sn-doped In${}_{2}$O${}_{3}$ (ITO), Al-doped ZnO (AZO) and F-doped SnO${}_{2}$ (FTO), which are degenerately doped n-type semiconductors. Highest electrical conductivities of ∼$10^{4}\;S/\mathrm{cm}$ are obtained with ITO, having carrier concentrations of 1–$2\times 10^{21}\;{\mathrm{cm}}^{-3}$ and mobilities of ∼$40\;{\mathrm{cm}}^{2}/\mathrm{Vs}$ [7]. Even higher conductivities are desirable, for example to reduce optical losses in solar cells by using thinner TCOs or wider cells in thin film modules. The conductivity can be increased either by a higher carrier concentration or a higher carrier mobility.

If thermodynamic equilibrium of defect concentrations can be established, the concentration of free electrons in TCOs is limited by the formation of self-compensating intrinsic defects [6,8,9,10]. In the case of donor-doped In${}_{2}$O${}_{3}$, the compensating defect species is interstitial oxygen [10,11,12,13,14,15]. Consequently, the diffusion of oxygen is required to establish defect equilibrium. At the limit of electrical conductivity, the addition of more donors to In${}_{2}$O${}_{3}$ does not result in an increase of the concentration of free electrons but in an increase of interstitial oxygen concentration. The carrier concentration can also be limited if the dopants are not completely dissolved in the material but form separate phases or segregate to grain boundaries and surfaces. Segregation requires mobile dopants. Both oxygen and dopant (Sn) diffusion in ITO have been demonstrated to occur already at $300{\;}^{\circ }C$ [16,17,18,19].

If samples are processed at temperatures low enough to suppress oxygen diffusion and dopant segregation, defect equilibrium cannot be established. In such a case, the concentration of compensating defects can, in principle, be lower than in equilibrium. Therefore, low processing temperatures of TCOs offer the potential advantage of achieving higher carrier concentrations. In contrast, in the case of donor-doped In${}_{2}$O${}_{3}$, conductivities of films deposited at lower temperature (≤$200{\;}^{\circ }C$) are typically lower than those of films prepared or annealed at higher temperature. In particular, films prepared at room temperature have conductivities below $10^{3}\;S/\mathrm{cm}$, mostly because of lower carrier concentrations [20,21,22,23,24]. Films prepared at room temperature are often amorphous. In this case, the carrier concentration does not depend on donor concentration but is rather determined by the oxygen stoichiometry [25,26]. Enhanced crystallization of room-temperature-deposited films has been achieved by using Fe${}_{2}$O${}_{3}$ seed layers [27]. Thereby, the carrier concentration can be enhanced by about one order of magnitude due to donor activation but remains below $10^{21}\;{\mathrm{cm}}^{-3}$.

Recently, Koida and coworkers have demonstrated that high conductivities of differently doped In${}_{2}$O${}_{3}$ films can be obtained by annealing room-temperature-deposited films to ∼$200{\;}^{\circ }C$ [28,29,30]. In this case, the addition of H${}_{2}$O during deposition can induce substantial grain growth during annealing and result in very high carrier mobilities of up to ∼$140\;{\mathrm{cm}}^{2}/\mathrm{Vs}$. However, even though the low temperature processing provides an advantage regarding temperature sensitive substrates and the high mobility enables an enhanced optical transparency in the infrared regime, the films still exhibit carrier concentrations lower than $10^{21}\;{\mathrm{cm}}^{-3}$. So far, there is no that evidence that carrier concentration above $2\times 10^{21}\;{\mathrm{cm}}^{-3}$ can be achieved by lower substrate temperatures.

As the conductivity of ITO thin films is determined by a number of factors, including crystallinity, grain size and oxygen content, which impact carrier concentration and mobility differently, it is difficult to discriminate between the different contributions. This becomes particularly important for the identification of the conditions needed to achieve higher carrier concentrations in low-temperature-processed samples.

In the present work, the effect of low processing temperature (≤$200\;{}^{\circ }C$) on the electrical properties of ITO thin films with different film thickness is studied. Together with in-operando Hall effect measurements, it becomes possible to discriminate between the effects of crystallization, grain growth, donor activation and oxygen diffusion on carrier concentrations and mobilities. The results provide guidelines for low-temperature processing of doped In${}_{2}$O${}_{3}$ films and will be used to explain the effects of Al${}_{2}$O${}_{3}$ deposition on the electrical properties of ITO and the conditions for realizing defect modulation doping of this compound. Defect modulation doping utilizes a Fermi level in a contact phase, which is pinned by defects at a high energy [31]. Carrier concentrations near an interface, which are higher than those observed by conventional doping, can be achieved by this technique. A suitable material with a high Fermi energy is obtained by low-pressure atomic-layer-deposited Al${}_{2}$O${}_{3}$ [32].

## 2. Experimental

ITO and Al${}_{2}$O${}_{3}$ films were prepared in the Darmstadt Integrated SYstem for MATerials research (DAISY-MAT), which combines several home-made deposition chambers with a multi-technique surface analysis system in a single ultrahigh vacuum cluster tool [6]. ITO films were deposited on quartz glass substrates by magnetron sputtering with radio-frequency (RF) excitation. The background pressure of the deposition chamber was $10^{-6}\;\mathrm{Pa}$. A ceramic 2 inch diameter ITO target with 10 wt% SnO${}_{2}$ doping, a RF power of 25 W, a process pressure of 0.5 Pa, an Ar flux of 6.6 sccm and a target-to-substrate distance of 10 cm were used for deposition. The film thickness of ITO was varied from 8 to 200 nm and the substrate temperature during deposition from room temperature to 400 ${}^{\circ }C$.

Al${}_{2}$O${}_{3}$ was deposited using a low-pressure ALD process in a separate vacuum chamber with a background pressure of $10^{-6}\;\mathrm{Pa}$ using Trimethylaluminium (TMA) from SAFC Hitech and purified water as precursors. The setup and Al${}_{2}$O${}_{3}$ deposition are described in detail in [32]. The ALD pulse lengths were set using ALD 3 series valves (Swagelock) with a microelectronic control unit to 80 ms for TMA and 150 ms for water. Pumping continued during exposure and each exposure was followed by pumping for 300 s, resulting in a total duration of an ALD cycle of 10 min. The growth of aluminum oxide was carried out at a substrate temperature of $200{\;}^{\circ }C$. We expect a homogeneous coverage of the ITO films by Al${}_{2}$O${}_{3}$ at the used thickness of 0.5 nm (5 cycles) due to (i) an exponential attenuation of XPS signals of the ITO substrate in dependence on ALD cycle number, (ii) an effective reduction of oxygen incorporation [32], and (iii) the successful preparation of capacitors with Al${}_{2}$O${}_{3}$ dielectric film thickness as low as 1.5 nm and electrodes of 100 μm diameter [31].

X-ray photoelectron spectroscopy (XPS) measurements were performed without breaking vacuum using a Physical Electronics PHI 5700 (Physical Electronics Inc., Chanhassen, MN, USA) spectrometer. Monochromatic Al Kα radiation with an excitation energy of 1486.6eV was used for XPS measurement. All spectra were recorded at an emission angle of $45{\;}^{\circ }$. The binding energies of the spectrometer were regularly calibrated by a sputter cleaned silver sample. Coplanar grazing incidence X-ray diffraction (GIXRD) patterns were collected with a Bruker D8 Advance Series II (Bruker AXS, Karlsruhe, Germany) and a Rigaku SmartLab (Rigaku, Tokyo, Japan) diffractometer in the 2θ range of 15–$85^{\circ }$ using Cu Kα radiation with a weighted wavelength of 1.54186Å. Room temperature and temperature-dependent Hall-effect measurements were carried out in van-der-Pauw geometry using a custom-made setup, which allows for continuous measurements with controlled temperature, pressure and gas composition [18,33].

## 3. Results and Discussion

### 3.1. Influence of Substrate Temperature and Film Thickness

#### 3.1.1. Microstructure of Room Temperature Deposited Films

**Figure 1:**
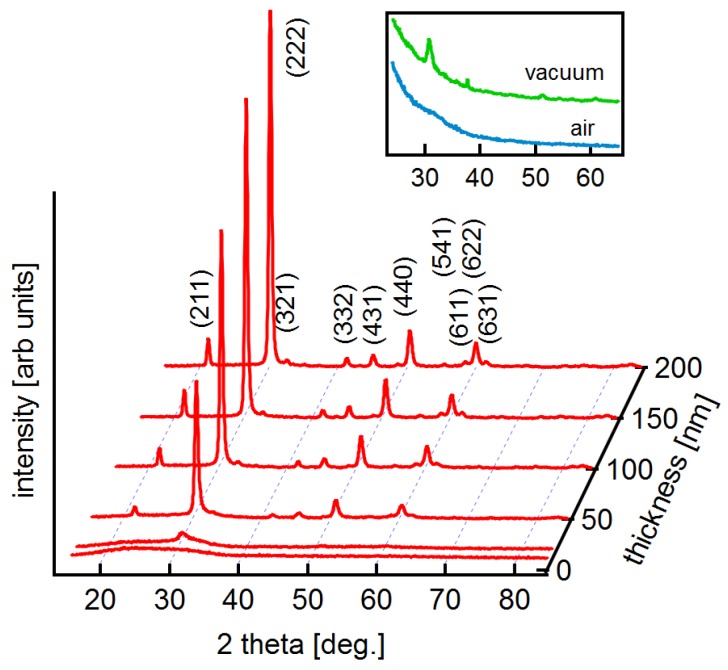
Grazing Incidence XRD patterns of ITO films with different film thickness deposited at room temperature. The indexed lattice planes correspond to those of cubic In${}_{2}$O${}_{3}$ (International Centre for Diffraction Data (ICDD) card 00-006-0416-High-bcc). The insert shows grazing incidence diffraction patterns of two 10 nm-thick films annealed at $200{\;}^{\circ }C$ in air or vacuum, respectively (see Section 3.2).

As a first step, the influence of substrate temperature on the electrical properties of ITO thin films with different thicknesses is presented in this section. Films deposited at elevated substrate temperature are expected to be crystalline but films grown at room temperature might be amorphous [20,25,34,35]. To verify this, grazing incidence X-ray diffraction has been performed of the films deposited at room temperature. The diffraction patterns are shown in Figure 1.

The thinnest film does not show any diffraction peaks. This is not due to the low film thickness, as clear peaks are observed after annealing the same film in vacuum (see insert; the effect of annealing is described in Section 3.2). With increasing film thickness, the diffraction peaks associated to In${}_{2}$O${}_{3}$ are increasing. Hence, only the thinnest film is completely amorphous after deposition. The increase of crystallinity with film thickness has also been reported in literature and can be attributed, on one hand, to the energy of the impinging particles [36] and, on the other hand, to an increasing substrate temperature induced by the heat of condensation of the film [20,35]. Nevertheless, despite the observation of crystalline structure, it is expected that the crystallite size of the films grown at room temperature is substantially smaller than that of films grown at higher temperature. The films may also still contain some amorphous regions. The microstructure is important for understanding the dependence of electrical properties on film thickness and during annealing as discussed below.

#### 3.1.2. Electrical Properties

**Figure 2:**
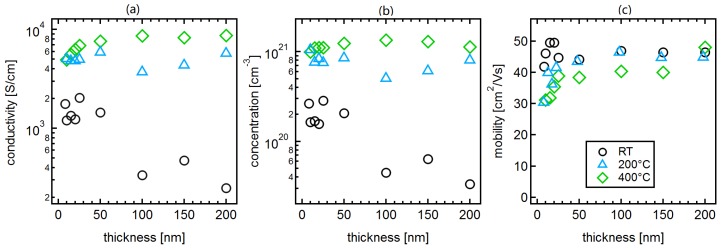
Electrical properties of ITO thin films deposited at different temperatures. Conductivity (**a**), carrier concentration (**b**) and carrier mobility (**c**) of ITO films deposited at room temperature, $200{\;}^{\circ }C$ and $400{\;}^{\circ }C$, as a function of film thickness.

The conductivities, carrier concentrations and carrier mobilities of films with thickness ranging from 10 to 200 nm, which are deposited either at room temperature, 200 or $400{\;}^{\circ }C$, are shown in Figure 2. The samples of each series are prepared consecutively within one week to keep changes of experimental conditions, such as the target consumption, as low as possible. The remaining deviation of data from straight trends, which is less than a factor of 2, might be caused by incomplete control of substrate temperature and film thickness, which are unavoidable in our setup due to in-situ sample transfer. The uncertainty of temperature and film thickness is typically less than 10%.

The carrier mobility is rather independent on film thickness and substrate temperature and exhibits values ∼$40\;{\mathrm{cm}}^{2}/\mathrm{Vs}$. The conductivity of the films generally increases with deposition temperature, whereby Hall effect measurements demonstrate that this is mostly due to an increase of carrier concentration.

In dependence on deposition temperature, the conductivity of the films exhibits a different evolution with film thickness. For films deposited at 400 ${}^{\circ }C$, the conductivity increases with film thickness, while the conductivity of films deposited at room temperature decreases with increasing film thickness. Due to the similar carrier mobilities, the different dependence on film thickness is related to changes of the carrier concentration. In the following, the dependence of carrier mobility and concentration on deposition temperature and film thickness will be discussed.

*Carrier mobility:* In general, the carrier mobility in highly doped TCO films is dominated either by ionized impurity scattering or by grain boundary scattering [19,37,38,39]. The latter is determined by the potential barrier height at the grain boundaries, $\phi_{B}$, according to:

(1) $$\mu =\mu_{0}\cdot \exp\left(-\frac{\phi_{B}}{k_{B}T}\right),$$

where $\mu_{0}$ is the mobility within the grains. In order to determine barrier heights quantitatively from temperature-dependent Hall effect measurements, one has to take the dependence of $\mu_{0}$ on temperature and carrier concentration into account [19]. These can be obtained from the fundamental scattering mechanisms [40]. Barrier heights in doped In${}_{2}$O${}_{3}$ thin films, which are determined using this technique, are <0.1 eV for carrier concentrations >$10^{18}\;{\mathrm{cm}}^{-3}$ [19]. It is mentioned that Equation (1) can only be applied if the space charge regions from neighboring grain boundaries do not overlap. This is very often not the case for the samples deposited at room temperature, as discussed below. As an example, the width of the space charge region is ∼5 nm for a doping concentration of $10^{20}\;{\mathrm{cm}}^{-3}$.

At the highest carrier concentrations of ∼$10^{21}\;{\mathrm{cm}}^{-3}$, the potential barriers at the grain boundaries are reduced and narrow enough for tunneling. The carrier mobility is then no longer reduced by the grain boundary potential barriers, but solely determined by ionized impurity scattering. In the case of highly Sn-doped In${}_{2}$O${}_{3}$, the electrons exhibit a carrier mobility of ∼$40\;{\mathrm{cm}}^{2}/\mathrm{Vs}$ [19,40]. This scenario explains that the carrier mobility of the films deposited at $200{\;}^{\circ }C$ and $400{\;}^{\circ }C$, which have carrier concentrations $5\times 10^{20}\;{\mathrm{cm}}^{-3}$ or higher, is largely independent on film thickness. The lower mobility of the thinner high-temperature-processed films is presumably related to a very small grain size (see also discussion of carrier concentration below).

**Figure 3:**
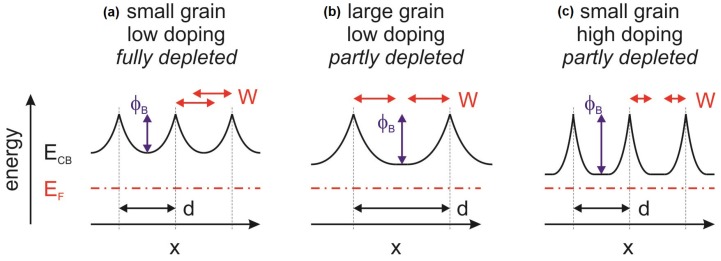
Energy band diagrams of n-type polycrystalline semiconductors with depleted electron concentrations in the space charge layers at grain boundaries. Positions of grain boundaries are indicated by dashed vertical lines. For small grains (**a**), when the width of the space region *W* exceeds half of the distance between neighboring grain boundaries (d<2W), the potential profiles of neighboring grain boundaries overlap as indicated by the red arrows. The width of the space charge region is ∼5 nm for a doping concentration of $10^{20}\;{\mathrm{cm}}^{-3}$. The potential barrier at the grain boundary, $\phi_{B}$, which corresponds to the band bending, increases with grain size (**b**) or doping concentration (**c**). Smaller grains can therefore, exhibit lower effective carrier concentrations and higher carrier mobility.

The situation is more complex for the films deposited at room temperature. The carrier mobility of the thinnest films, which are amorphous, correspond well with those of amorphous ITO films reported in literature [26,27,34]. Hoewever, the films start to crystallize with increasing film thickness and the carrier concentration is reduced to values as low as ∼$4\times 10^{19}\;{\mathrm{cm}}^{-3}$. It is therefore, expected that the carrier mobility should decrease with increasing film thickness due to grain boundary scattering. For example, polycrystalline films deposited at $400{\;}^{\circ }C$ have mobilities of only ∼$15\;{\mathrm{cm}}^{2}/\mathrm{Vs}$ for carrier concentrations of $4\times 10^{19}\;{\mathrm{cm}}^{-3}$ [19]. In contrast to this, the mobility of the room-temperature-deposited films remains as high as that of films with higher carrier concentration. This unexpected high mobility of room-temperature-deposited crystalline films with carrier concentrations <$10^{20}\;{\mathrm{cm}}^{-3}$ is suggested to be related to a small grain size. If the grains are very small, the depletion regions induced by the potential barriers at adjacent grain boundaries overlap and the bending of the energy bands inside a grain will consequently be reduced as illustrated in the left graph of Figure 3. The potential barrier for grain boundary scattering is then reduced according to the Seto model, in which the potential barrier is equivalent to the band bending [37,38].

*Carrier concentration:* The carrier concentration of films deposited at 400 ${}^{\circ }C$ is reduced at low film thickness, compared to that of thicker films. This can be explained by a thickness dependent change in grain size, which is common in film growth. In the presence of potential barriers at grain boundaries, the average carrier concentration, $\bar{n}$, is determined by the integral of the local carrier concentration according to the expression:

(2) $$\bar{n}=\frac{1}{d}\int_{0}^{d}n\left(x\right)dx=\frac{1}{d}\int_{0}^{d}\int_{E_{\mathrm{CB}}\left(x\right)}^{\infty }N_{C}\left(E\right)\frac{1}{1+e^{\left(E-E_{F}\right)/k_{B}T}}dE\;dx$$

where $N_{C}\left(E\right)$ is the density of states in the conduction band [41]. The average carrier concentration decreases when the grains become very small as in the initial stage of growth (see Figure 3).

The carrier concentration of films deposited at room temperature decreases with increasing film thickness. This behaviour must be determined by a combination of effects as the carrier concentration in the thinner, amorphous films is determined by the coordination of the cations with oxygen, while the Sn-dopants do not contribute to the carrier concentration [25,26]. The dopants only contribute to the carrier concentration in crystalline films. Therefore, it is expected that the carrier concentration increases once the films crystallize with increasing film thickness. The opposite is observed in Figure 2. The decrease of carrier concentration with film thickness can be explained if it is assumed that the grain size of the thicker (crystalline) films, grown at room temperature, remains very small. The depletion regions of adjacent grain boundaries will then overlap leading to a decrease of average carrier concentration (see Figure 3). This is believed to be the main reason for the lower carrier concentration of thick films deposited at room temperature as compared to films grown at higher temperature. In addition, a higher oxygen incorporation into the films during deposition at room temperature may also contribute to the lower carrier concentration. It is reasonable to assume that less oxygen is incorporated at higher deposition temperature as the residence time of oxygen on the growing film’s surface decreases with temperature. Moreover, higher temperatures generally correspond to more reducing conditions due to the temperature-dependence of the oxygen chemical potential [42].

### 3.2. The Effect of Annealing

**Figure 4:**
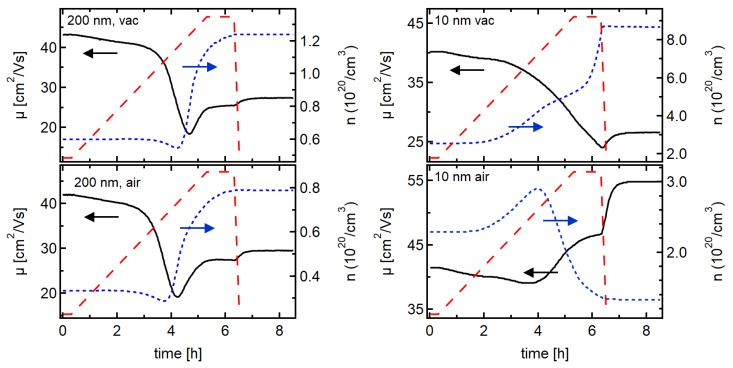
Hall effect measurements during annealing of 10 (right)and 200 (left) nm-thick ITO films deposited at room temperature in vacuum (top) or in air (bottom). The red dashed lines show the programmed temperature with a controlled heating ramp from 25–200 ${}^{\circ }C$ and a holding time of 1 h at 200 ${}^{\circ }C$.

It has been suggested in the previous section that the low electrical conductivity of thicker films, deposited at room temperature, is mostly determined by their microstructure, which is assumed to be characterized by a very small grain size. In order to confirm this, in-operando Hall effect measurements have been conducted during annealing of films deposited at room temperature. In order to follow the changes in conductivity, carrier concentration and mobility, samples were heated either in vacuum ($10^{-5}\;\mathrm{Pa}$) or in air with a rate of 0.5K/min up to $200{\;}^{\circ }C$ and kept at that temperature for 1 h. The results obtained for ITO films of 10 and 200 nm thickness are shown in Figure 4.

The changes during the heat treatment in vacuum or in air are almost identical for the 200 nm-thick films. Only the magnitude of the carrier concentration is slightly different regarding the two studied samples. For both samples, the carrier concentration increases by about a factor of 2 and the carrier mobilities decrease first by a factor of 2 before they slightly increase again. Both carrier concentration and mobility saturate after 1 h at $200{\;}^{\circ }C$.

In contrast to the 200 nm-thick films, the annealing behaviour of the 10 nm-thick films is drastically different for annealing in vacuum and air. Annealing in vacuum results in a behaviour similar to that of the 200 nm-thick films, with an increasing carrier concentration and a reducing mobility. However, in contrast to the thicker films, the carrier concentration and the mobility do not saturate after 1 h at $200{\;}^{\circ }C$ and the carrier concentration increases much more. In contrast to the vacuum anneal, the carrier concentration of the 10 nm-thick film decreases after an initial increase during annealing in air. Concurrently, the mobility increases.

The annealing atmosphere has also an effect on the crystallization behaviour of the 10 nm films. Grazing incidence X-ray diffraction, which is shown in the insert of Figure 1, only shows diffraction peaks after annealing in vacuum. The film annealed in air remains amorphous. How the different oxygen content affects crystallization is unclear and has to be subject of further studies.

The different crystallinity of the 10 nm-thick films after annealing in air or in vacuum is also reflected in a different change of carrier mobility at the end of annealing (see Figure 4). The air annealed, amorphous film shows a much higher increase in mobility during cooling down from $200{\;}^{\circ }C$ to room temperature than the other three samples. Such an increase of μ with decreasing temperature is expected for ionized impurity scattering. A lower or even inverted temperature-dependence indicates the presence of grain boundary scattering [19] and therefore, the presence of a crystalline structure. Grain boundary scattering should not be present in amorphous materials, which is consistent with the temperature-dependence of the mobility of the air annealed 10 nm film.

In the following, the evolution of electrical properties during annealing is discussed separately for the 200 nm and the 10 nm-thick films.

#### 3.2.1. 200 nm-Thick Films

The comparable behaviour of the 200 nm-thick films upon annealing in vacuum and air indicates that the changes in carrier concentration are not related to changes of the oxygen interstitial content of the films as such changes should depend on the annealing atmosphere. It is pointed out that the change of oxygen content of a film includes two steps [42,43]: (i) the oxygen surface exchange, characterized by the surface exchange coefficient *k* (in units of m/s) and (ii) the diffusion of oxygen in the film, characterized by the diffusion coefficient *D* (in units of m${}^{2}$/s). Either of the two can be the rate determining step. The fact that the oxygen content of the 200 nm films is not changing at $200{\;}^{\circ }C$ is consistent with DFT calculations of oxygen diffusion [17] and with in-operando Hall effect measurements of crystalline ITO films [18], indicating that oxygen diffusion at $200{\;}^{\circ }C$ is not fast enough to substantially change the electrical properties within a few hours of annealing time.

The increase in carrier concentration and the decrease of mobility during heat treatment of the 200 nm-thick films in either atmosphere, can both be explained by crystallization and grain growth. Crystallization of amorphous regions, which might still be present in the 200 nm-thick films deposited at room temperature, would activate the Sn donors [25,26] and thereby increase the carrier concentration. Consequently, the increased carrier concentration reduces the width of the space charge region. The overlap of space charge regions from neighbouring grain boundaries will then be reduced and the band bending within the grain will be increased. The situation is illustrated in Figure 3. The reduced overlap of space charge regions results in an increase of the average carrier concentration. The concomitant increase of $\phi_{B}$ explains the observed decrease of carrier mobility. In addition to an increase of dopant concentration by donor activation, grain growth would also result in an increase of carrier concentration and in a reduction of mobility. This is also consistent with the changes of electrical properties of the 200 nm-thick films upon annealing. Therefore, both donor activation and grain growth may contribute to the observed changes in electrical properties.

#### 3.2.2. 10 nm-Thick Films

*Vacuum annealing:* The substantial increase of carrier concentration during vacuum annealing of the 10 nm-thick film indicates a substantial increase in effective dopant concentration. According to the discussion above, two different effects can contribute to this observation: (i) donor activation by crystallization and grain growth; (ii) extraction of oxygen. Crystallization during vacuum annealing is clearly demonstrated by the GIXRD results in the insert of Figure 1. Large grain sizes are not expected, given the moderate annealing temperature of only $200{\;}^{\circ }C$. Crystallization alone can therefore, hardly account for the large increase in carrier concentration.

The annealing of the 200 nm-thick films indicates that a change of the overall oxygen interstitial content in the film is not important for the 200 nm-thick films. However, it can be important in thin films as the time τ required to establish equilibrium by bulk diffusion (of oxygen) depends on the square of the film thickness *L* and is given by $\tau =L^{2}/\pi^{2}D$, where *D* is the diffusion coefficient [44]. Given this dependence, it is not unlikely that a change of oxygen content can contribute to the changes of carrier concentration at 200 ${}^{\circ }C$ for 10 nm-thick films but not for thicker ones. If, as argued above, oxygen diffusion is the time limiting step for the change of the oxygen content in the film, one can furthermore conclude that the oxygen surface exchange is not. This is in agreement with previous studies [18].

*Air annealing:* The decrease of carrier concentration and increase of mobility during air annealing of the 10 nm-thick film can also be explained by a change of oxygen content. Medvedeva and coworkers have demonstrated that the carrier concentration in amorphous In${}_{2}$O${}_{3}$ films is determined by the oxygen stoichiometry [26]. Incorporation of oxygen will reduce the effective donor concentration and thereby reduce the carrier concentration. This will reduce the amount of scattering centers and thereby raise the mobility, in agreement with the observation. Island formation as origin of the reduced carrier concentration is not expected for the moderate annealing temperature. This agrees with AFM measurements of ITO films with comparable thickness reported by Sytchkova and coworkers [45].

#### 3.2.3. Extended Annealing in Vacuum

**Figure 5:**
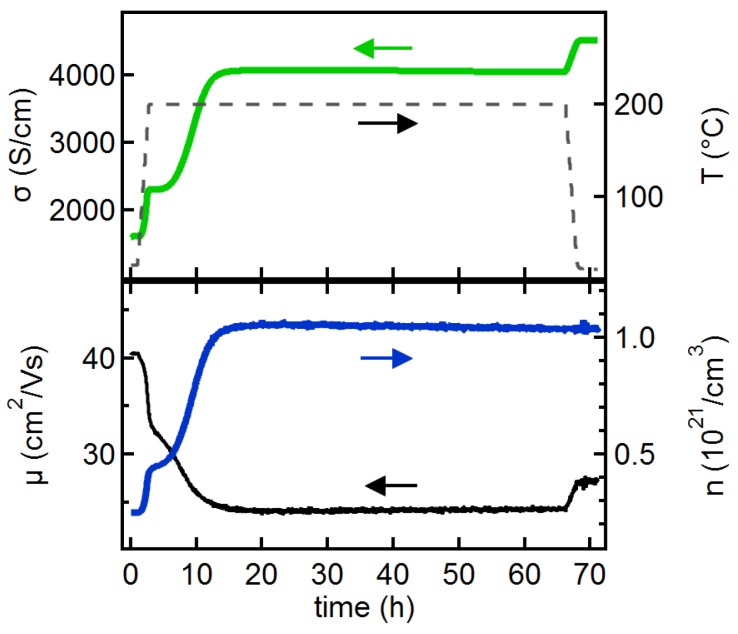
Conductivity and Hall effect measurements during extended annealing of a 10 nm-thick ITO film deposited at room temperature in vacuum.

To further check whether the removal of oxygen contributes to the strong increase of carrier concentration of the 10 nm film during vacuum annealing, an extended annealing experiment has been carried out. The measurement is shown in Figure 5.

At the beginning of the annealing, the behaviour observed in Figure 5 compares well with that observed during vacuum annealing of the 10 nm-thick film shown in Figure 4. In the experiment shown in Figure 5, the carrier concentration and mobility saturate after ∼15h annealing at $200{\;}^{\circ }C$. The saturated carrier concentration amounts to $n\approx 1.05\times 10^{21}\;{\mathrm{cm}}^{-3}$, which is higher than those observed for deposition at $200{\;}^{\circ }C$ (see Figure 2). After cooling down to room temperature, a conductivity of 4520 S/cm is reached. This is of the same magnitude as those obtained with films of the same thickness at higher deposition temperatures. Vacuum annealing is therefore, suitable to obtain very high conductivities of very thin films. However, the required annealing times are rather long. Annealing experiments with fixed (shorter) annealing times and post-anneal analysis can therefore, only provide a snapshot of the annealing effects. This can make an analysis of the origin of the changes induced by annealing from such experiments difficult.

The very high carrier concentrations reached after long vacuum annealing of the 10 nm film, can only be reached if oxygen is extracted from the films during annealing. This can be concluded by comparison with ITO films grown under identical process conditions in our laboratory at room temperature but on Fe${}_{2}$O${}_{3}$ seed layers [27]. The Fe${}_{2}$O${}_{3}$ seed layers strongly enhance crystallization at room temperature, resulting in a substantial increase of carrier concentration due to donor activation and increased grain size. The carrier concentrations reached with Fe${}_{2}$O${}_{3}$ seed layers are <$6.5\times 10^{20}\;{\mathrm{cm}}^{-3}$. Therefore, donor activation by crystallization is not sufficient to explain the carrier concentrations higher than $10^{21}\;{\mathrm{cm}}^{-3}$ reached in this work by vacuum annealing.

**Figure 6:**
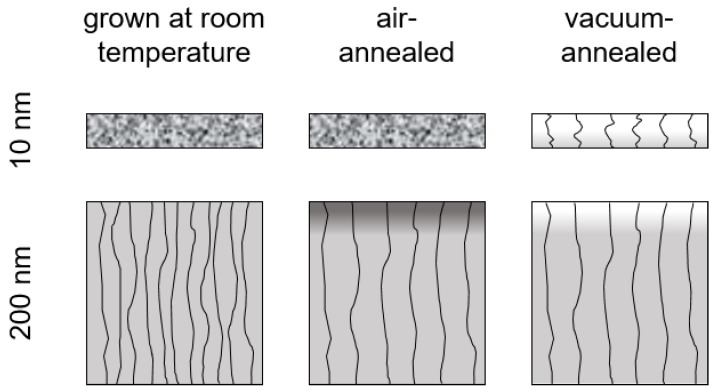
Effect of annealing of 10 and 200 nm-thick ITO films deposited at room temperature. The annealing was performed with a heating rate of 0.5K/min up to $200{\;}^{\circ }C$ and a holding time of 1 h either in vacuum or in air. Curved lines indicate grain boundaries and filling color the oxygen concentration. The filling style of the as-grown and the air-annealed 10 nm-thick sample indicates their amorphous structure.

The effect of annealing in dependence on film thickness and atmosphere is summarized in Figure 6.

### 3.3. Effect of Al${}_{2}$O${}_{3}$ Deposition

#### 3.3.1. XPS Analysis

**Figure 7:**
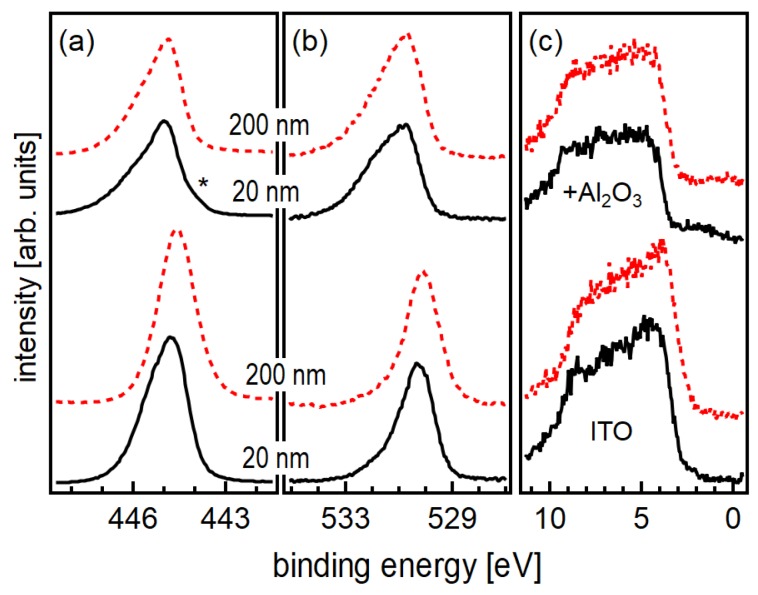
In 3d (**a**), O 1s (**b**) and valence band (**c**) X-ray photoelectron spectra of 20 nm (solid black lines) and 200 nm (dashed red lines) thick ITO films before (bottom) and after (top) deposition of Al${}_{2}$O${}_{3}$ using 5 ALD cycles. The asterisk indicates a shoulder in the In 3d spectrum, which corresponds to a partial reduction of In.

X-ray photoelectron spectra of 20 and 200 nm-thick ITO films deposited at room temperature before and after 5 ALD cycles of Al${}_{2}$O${}_{3}$, corresponding to an Al${}_{2}$O${}_{3}$ thickness of 0.5 nm, are shown in Figure 7. The binding energies of the uncoated 20 nm-thick film are higher than those of the uncoated 200 nm-thick film. This corresponds well with the higher carrier concentration of the thinner film as discussed in Section 3.1.

Deposition of Al${}_{2}$O${}_{3}$ results in the appearance of Al 2p and Al 2s emissions (not shown) and a reduction of the intensities of the In 3d core level and the valence band emission. After Al${}_{2}$O${}_{3}$ deposition, the O 1 s line exhibits a pronounced additional emission at a binding energy of ∼532eV, which can be assigned to oxygen in the Al${}_{2}$O${}_{3}$ layer. In agreement to previous work [32], in which the Al${}_{2}$O${}_{3}$ films were grown by ALD onto ITO films deposited at 400 ${}^{\circ }C$, the binding energies of the ITO substrate emissions are increased by Al${}_{2}$O${}_{3}$ deposition. This corresponds to a rise of the Fermi energy and indicates a surface electron accumulation. Such accumulation layers have been frequently reported to be present at In${}_{2}$O${}_{3}$ surfaces [46,47,48,49]. We like to point out that no accumulation layer is present on the as-deposited surfaces, as the samples have not been exposed to air before XPS measurement [6].

The Fermi energy in the Al${}_{2}$O${}_{3}$ films deposited by the low-pressure process in the DAISY-MAT system is reproducibly pinned at 4.5eV above the valence band maximum, independent on substrate [31,32,50,51]. Together with a very small valence band offset between ITO and Al${}_{2}$O${}_{3}$ [32,52], it is expected that the Fermi energy in the ITO also raises to $E_{F}-E_{\mathrm{VB}}>4\;\mathrm{eV}$. In contrast, the binding energies of the In 3d core level do not correspond to such high Fermi energies but only to $E_{F}-E_{\mathrm{VB}}\approx 3.3\;\mathrm{eV}$ [16]. This too low binding energy of the substrate emission could be explained by the formation of a very narrow space charge region at the surface of the highly doped ITO, which is narrower than the depth probed by XPS. This is equivalent to an effective modification of the band alignment [6,32,53,54]. However, the upward shift of the Fermi energy after Al${}_{2}$O${}_{3}$ deposition is also partially related to a chemical reduction of the substrate. An Al${}_{2}$O${}_{3}$-deposition induced reduction of the 20 nm-thick film is indicated in Figure 7 by the appearance of a shoulder on the low binding energy side of the In 3d emission and from the increased band gap emission in the valence band spectrum.

In contrast to the results reported for SnO${}_{2}$ [31], the XPS analysis does therefore, not provide evidence for a modulation doping effect at the ITO/Al${}_{2}$O${}_{3}$ interface.

#### 3.3.2. Electrical Analysis

**Figure 8:**
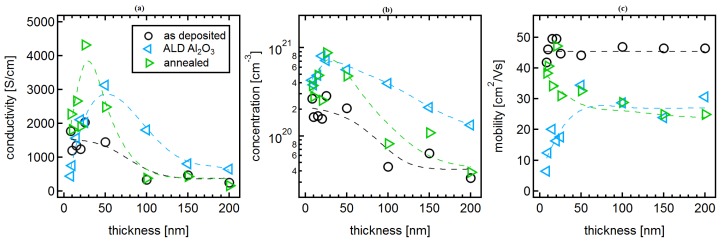
Hall effect measurements as a function of film thickness of ITO films deposited at room temperature (black circles), ITO films deposited at room temperature after Al${}_{2}$O${}_{3}$ deposition, and ITO films deposited at room temperature after annealing in the ALD chamber (ALD-anneal) in vacuum at 200 ${}^{\circ }C$. Conductivity (**a**), carrier concentration (**b**) and carrier mobility (**c**). The data of the as-deposited films are the same as those in Figure 2. Dashed lines are a guide for the eye.

Conductivity and Hall effect measurements, performed on ITO films deposited at room temperature, are shown in Figure 8, together with those obtained after Al${}_{2}$O${}_{3}$ deposition. Data for uncoated samples are the same as those shown in Figure 2.

The Al${}_{2}$O${}_{3}$ deposition is performed in a vacuum chamber at $200{\;}^{\circ }C$ and involves heating of the samples in vacuum before exposure to the process gas. As indicated by the results described in Section 3.2, the heating process in vacuum can already affect the electrical properties of room-temperature-deposited films via oxygen extraction (low film thickness) and grain growth (thicker films). To discriminate between the effects of temperature and Al${}_{2}$O${}_{3}$ deposition, additional samples were annealed in the ALD chamber under the same conditions present during the ALD process, just without exposure to TMA and H${}_{2}$O. This annealing, which is referred to as ALD-anneal here, is shorter than the one performed in the Hall effect setup. Conductivity and Hall effect measurements performed after the ALD-anneal are included in Figure 8. In the following, the effects of the ALD-anneal and the Al${}_{2}$O${}_{3}$ deposition on the carrier mobility and concentration are described and discussed.

*Carrier mobility:* As already discussed in Section 3.1, the mobility of room-temperature-deposited films is independent on film thickness due to combined effects of crystallization (donor activation) and grain size. The effect of the ALD-anneal is comparable to that described for vacuum annealed films in Section 3.2. In particular, the mobility is not affected for thinner films but decreases to ∼$25\;{\mathrm{cm}}^{2}/\mathrm{Vs}$ for films thicker than 30 nm. Al${}_{2}$O${}_{3}$ deposition results in a reduction of mobility for all film thicknesses. The mobility after Al${}_{2}$O${}_{3}$ deposition is comparable to the mobility of ALD-annealed samples for samples thicker than 30 nm. For thinner films, Al${}_{2}$O${}_{3}$ deposition reduces the mobility substantially.

As the reduction of mobility of Al${}_{2}$O${}_{3}$-coated films thicker than 30 nm is equivalent to that of the ALD-annealed films, the changes in mobility are evidently caused by the temperature treatment. Consequently, the mobility is mostly determined by the effects of grain growth, as described in Section 3.2. In contrast, the strong reduction of mobility of the thinner Al${}_{2}$O${}_{3}$-coated films is not related to the temperature treatment but must be caused by the ALD deposition. It might be related to the chemical reduction of the film, which is evident from XPS (see Figure 7). The reduction will increase scattering centers and/or increase grain boundary potential barriers by reduction of Sn to Sn${}^{+\mathrm{II}}$ [19]. Both effects will reduce the mobility. As the reduction is restricted to the surface region due to the limited diffusivity of oxygen at $200{\;}^{\circ }C$, the observed increase of mobility of the Al${}_{2}$O${}_{3}$-coated films with ITO thickness appears reasonable, as the mobility is only reduced in the near surface region while the measured mobility will be dominated by the interior of the film.

*Carrier concentration:* Independent on film thickness, the carrier concentration is increased by Al${}_{2}$O${}_{3}$ deposition. The highest carrier concentrations of $n\approx 8\times 10^{20}\;{\mathrm{cm}}^{-3}$ are obtained for 20 and 25 nm-thick films. For a film thickness of up to 50 nm, the increase of carrier concentration by Al${}_{2}$O${}_{3}$ deposition is identical to that induced by the ALD-anneal. Thicker ALD-annealed films have carrier concentrations only slightly higher than those of as-deposited films. Thicker films exhibit higher carrier concentrations after Al${}_{2}$O${}_{3}$ deposition than after the ALD-anneal.

Due to the comparable dependence and numbers, the Al${}_{2}$O${}_{3}$ deposition induced increase of the carrier concentration of films up to 50 nm thickness can be ascribed to the temperature treatment and not to the Al${}_{2}$O${}_{3}$ deposition. This again excludes a modulation doping effect as this should only be observed after Al${}_{2}$O${}_{3}$ deposition. The increase in carrier concentration of the thinner films is therefore, likely caused by donor activation due to crystallization combined with a removal of oxygen from the films as discussed in Section 3.2. The only small increase of carrier concentration of thicker films is consistent with the results from vacuum annealing of thicker films, which have been described in Section 3.2.

For films thicker than 50 nm, the Al${}_{2}$O${}_{3}$-coated films exhibit higher carrier concentrations than the ALD-annealed ones. Apparently, the exposure to Al${}_{2}$O${}_{3}$ is more reducing than vacuum heating at $200{\;}^{\circ }C$, in agreement with the partial reduction of the films observed by XPS. In comparison to the less reducing vacuum annealing, the Al${}_{2}$O${}_{3}$ deposition may not only result in extraction of more oxygen but also in an enhanced grain growth. Both effects might contribute to the higher carrier concentration after Al${}_{2}$O${}_{3}$ deposition.

*Absence of defect modulation doping:* In contrast to SnO${}_{2}$ [31], Al${}_{2}$O${}_{3}$ deposition onto ITO does not induce modulation doping. According to the results described in this work, this is very likely related to the high mobility of oxygen defects. The annealing experiments discussed in Section 3.2 demonstrate that oxygen defects are sufficiently mobile to diffuse several nanometers during processing at $200{\;}^{\circ }C$ during the employed Al${}_{2}$O${}_{3}$ process. As a consequence, compensating oxygen interstitial defects will diffuse towards the interface in response to a raising Fermi energy and screen the potential difference [55]. The fundamental condition required to enable defect modulation doping, which is the kinetic suppression of equilibrium defect distribution, can therefore, not be met with ITO at $200{\;}^{\circ }C$. Lower processing temperatures for ALD deposition would be required. Such lower temperature ALD processes have been demonstrated in literature [56,57].

## 4. Summary and Conclusions

Low-temperature-processed ITO thin films offer the potential of overcoming the doping limit by suppressing the equilibrium of compensating oxygen interstitial defects. The aim of this work was to provide a more detailed understanding of the processes, which determine the carrier concentrations of ITO films processed at substrate temperatures, at which oxygen diffusion and cation segregation is suppressed. For this purpose, electrical properties of Sn-doped In${}_{2}$O${}_{3}$ thin films as a function of film thickness have been presented and discussed. Films deposited at room temperature exhibit significantly lower conductivities compared to films deposited at $200{\;}^{\circ }C$ and $400{\;}^{\circ }C$. The differences are caused by different carrier concentrations, while the mobilities are rather insensitive on deposition temperature and film thickness.

Only the thinnest films deposited in our setup at room temperature are completely amorphous. The intensity of diffraction peaks increases with increasing film thickness, which has been assigned to a slow increase of substrate temperature with deposition time and to ion bombardment effects.

The carrier concentration of the room-temperature-deposited films decreases with film thickness. As the carrier mobility is not affected by the reduced carrier concentration, which would be expected for grain boundary scattering, the reduction of *n* has been assigned to the formation of very small grains with overlapping space charge regions.

Room temperature-deposited amorphous 10 nm-thick and crystalline 200 nm-thick films have been annealed either in vacuum or in air at $200{\;}^{\circ }C$. Conductivity and Hall effect measurements were recorded during the complete annealing cycles. The thicker films exhibit a slight increase in carrier concentration and reduction of mobility during annealing, regardless of the annealing atmosphere. The changes can be explained by grain growth. A change of effective doping concentration by variation of the oxygen content is not indicated. This is consistent with previous experiments and calculations on oxygen diffusivity.

In contrast to the 200 nm-thick films, the evolution of electrical properties of the 10 nm-thick films inevitably involves a change of oxygen concentration, indicating that oxygen diffusivity (and oxygen surface exchange) is fast enough for 10 nm-thick films. Therefore, extraction of oxygen during annealing in vacuum results in a substantial increase of carrier concentration by almost an order of magnitude up to $n>10^{21}\;{\mathrm{cm}}^{-3}$, while incorporation of oxygen during annealing in air results in a decrease of carrier concentration. It is noted that, in contrast to annealing in vacuum, annealing in air does not lead to crystallization of the 10 nm-thick films. The differences might be related to the different changes of oxygen content in dependence on annealing atmosphere.

During annealing at $200{\;}^{\circ }C$, diffusion of oxygen does, therefore, only affect a very thin region near the surface of thicker films. Manipulation of the carrier concentration in thicker films is therefore, not an option at this temperature. The remaining diffusivity of compensating oxygen defects at $200{\;}^{\circ }C$ is, however, sufficient to screen the high Fermi level induced by deposition of Al${}_{2}$O${}_{3}$ using atomic layer deposition. At this temperature, defect modulation doping can therefore, not be applied to ITO. However, the growth of Al${}_{2}$O${}_{3}$ at lower substrate temperatures remains an option for defect modulation doping.

Finally, realization of highest carrier concentrations in ITO thin films should be possible by using a low-substrate temperature-deposition process with a very low oxygen activity. Nevertheless, the inherently small grain size of low-temperature grown films has to be overcome, as this clearly limits the carrier concentration. The use of seed layers, such as the recently demonstrated Fe${}_{2}$O${}_{3}$ [27], might be a solution for this.

## Abbreviations

The following abbreviations are used in this manuscript:

ITO	Sn-doped In${}_{2}$O${}_{3}$
FTO	Fluorine-doped SnO${}_{2}$
AZO	Al-doped ZnO
ALD	atomic-layer-deposition
TMA	trimethylaluminium
XPS	X-ray photoelectron spectroscopy
XRD	X-ray diffraction
GIXRD	grazing incidence X-ray diffraction
RF	radio frequency
